# Analecta

**Published:** 1842-08-20

**Authors:** 


### ANALECTA.

*Complete Dislocation of the Tibia backwards.*—C. B. Rose, Esq., of Swaffham, narrates the following case of this rare accident in a communication to the Provincial Medical Journal for June 11th, 1842.

On the 17th of December, 1829, I was hastily summoned to an accident which had occurred at the principal inn of this town, situated within fifty yards of my own door: a stage coach had been just driven up to change horses, and, as is usual, a ladder placed beside and against it; the woman who collected and delivered the parcels had ascended the ladder, and was standing upon it, when a man in a state of intoxication drove furiously against the ladder, upsetting it and throwing the woman violently to the ground; she was immediately taken up, and carried into the kitchen of the inn; I was quickly by her side, and on examining the injured limb, found the tibia completely dislocated at the knee, the head of that bone having been driven behind the condyles of the femur into the ham, with the patella thrown to the outside of the external condyle of the femur, and the leg in a state of fixed extension.

I immediately, and without difficulty, restored the parts to their normal situation, by applying one hand to the patella, the other to the back part of the upper portion of the tibia, and simultaneously pulling and pushing those bones into their natural positions. The patient was then removed to her home, a distance of about two hundred yards from the inn, and placed in bed. By the employment of leeches, evaporating lotions, and strict rest, inflammation was kept in check; in short, no untoward symptoms whatever arose, and after the lapse of a few weeks she perfectly recovered the use of the joint.

[*Remarks.*—The writer of the article refers to an equally successful case of dislocation forwards, by Mr. Coote, narrated in a paper read before the Royal Medical and Chirurgical Society, April 26th, 1842, and noticed in the Provincial Journal for May 14th. Mr. C. opposed the idea that the question of amputation was *necessarily* involved in such cases, or that the recovery of perfect motion in the joint was hopeless, citing in support of his own experience the caution against hastily operating recommended by Delpech. The paper is reported as entering "at some length into the anatomical examination of the ligaments of the joint, for the purpose of showing that dislocation may take place without any material laceration of them."

Mr. R. disproves the supposition of Sir Astley Cooper, (in discussing a case seen by Dr. Walshman,) that this accident must produce a detachment of the patella from the rectus or from the tibia. He also quotes from Wiseman another instance of the recovery of the motions of the knee-joint after this accident. M. Boyer represents the position of the leg as that of very acute flexion; and Mr. Rose refers to his own case, together with that of Dr. Walshman, (the latter having given origin to the description of the characters of the accident as drawn by Sir Astley, which represents the leg as bent forwards,) seemingly in proof of the inaccuracy of M. Boyer's account. But we are inclined to think that each of the positions mentioned is equally possible, the attitude of the limb being obviously capable of modification by the nature and direction of the dislocating force, and the extent and character of the muscular or ligamentous lacerations. Mr. Wiseman succeeded in accomplishing the reduction by a sudden and complete flexion of the leg after gentle extension—a practice which would be warrantable—if at all—only after the failure of all other measures. In the other cases mentioned, the reduction of the tibia, and the patella also, when displaced, appears to have been very easily effected by the most obvious and simple processes.]

R. C.

---

*On the employment of the Sulphate of Alum in the treatment of some forms of Angina Pharyngea.* By M. Celestin Perrin.—It is by no means unusual for catarrhal affections, especially in damp situations, to leave behind them a sort of habitual chronic catarrh of the fauces. In these cases the mucous membrane is much injected, of a deep red, sometimes thickened, and the mucous follicles are very apparent and much developed. An adhesive mucus covers the parts and provokes a frequent and troublesome cough to effect its expectoration. The employment of alum gargles, of various strength, in these affections has for some years been often resorted to. M.

Petrèquin, of the Hôtel Dieu, has practised the insufflation of four parts of alum to one of sugar with great success; and M. Perrin has used the same means with similar results.

Encouraged by the good effects of the application in chronic cases, M. Perrin has had recourse to it in those which are acute. He mixes equal parts of alum and sugar, and blows them through a quill against the back of the pharynx. It is always necessary that the point of the quill should be even with the uvula, since otherwise the sudden descent of the velum palati may close the passage and scatter the powder on the back of the tongue, where it excites nausea and efforts at vomiting. Even in cases where the febrile symptoms run very high, the difficulty of swallowing is extreme, and the patients have on former occasions been depleted and subjected to very severe treatment, this application a few times repeated has seemed to effect a cure, and a great amelioration of the symptoms has followed its employment even once. Two cases are related in illustration, and the writer concludes by asking whether equally favourable results might be expected from this practice in cases occurring in dry and hot countries, or whether there is something peculiar in the anginas of damp and rainy climates, as Lyons, which renders them peculiarly amenable to this mode of treatment.—*British and Foreign Medical Review. July*, 1842. *From Bulletin Général de Thérapeutique.* Mars, 1842.

---

***On the Delirium following Acute Meningitis, and its Treatment by Cold Affusion.*** By M. Recamier.—In the Hôtel Dieu, under the care of M. Recamier, was a man who was the subject of chronic delirium, unaccompanied by any sign of disease of the brain or its membranes. He had already had two attacks of a similar kind, the second more severe than the first, and the third than the second, but they all yielded to depletion, which in the last instance required to be repeated several times. In the same ward was another patient in whom delirium remained after symptoms of inflammation of the brain, and depended, in M. Recamier's opinion, on some modification in innervation produced by the disease, not on any continuance of the actual malady.

Two other similar cases are mentioned by him in which chronic delirium was removed by the employment of cold affusion. One was the case of a young lady in whom delirium existed unaccompanied by the signs of meningitis, but with irregular automatic movements of the hands, in which she crammed anything that was given her into her mouth. M. Recamier began the treatment with cold affusion of water at 68° Fahrenheit during five minutes, and gradually reduced the temperature to 50°, and prolonged the affusion for ten minutes. It was not, however, until affusion was practised at 39° that any effect was produced, but then the patient was seized with a tetanic spasm, and lost all consciousness. She was at once placed in bed, warmth was applied to the surface, and she speedily recovered, and asked for her mother, for whom she had before shown a marked aversion. Pen and paper being given her, she now wrote a perfectly sane letter, requesting her mother to visit her. The affusions were still continued at a temperature of about 64° for some days, and the patient recovered uninterruptedly.

In this case, which had followed an attack of meningitis, warm baths had been employed for a considerable time, without leading to any results. In the second case the same treatment was employed with success in a man who had become insane from spirit-drinking. In three different attacks the remedy had the effect of restoring his reason, but his intemperate habits were so deeply rooted that he eventually brought on a condition of permanent delirium, for which he was received into the Bicêtre.—*Ibid., from Gazette des Hôpitaux.* Mars 3, 1842.

---

*Extemporaneous Production of Milk.* By M. DICHOST.—M. Dichost, a Russian chemist, proposed the following plan for the preservation and extemporaneous preparation of milk. He evaporates newly-drawn milk, at a very gentle heat, till it is all brought to a state of fine powder. It is then put into small glass bottles, which are completely filled and hermetically sealed, with ground glass stoppers. A small quantity of the powder thus obtained, dissolved in an appropriate quantity of water, affords on the instant a milk of very good quality. The powder will remain good for a great length of time.—*Ibid. from Ibid.* Janvier 22, 1842.

---

*On the External Application of Croton Oil.* By M. BOUCHARDAT.—Whenever it is required to use this method of counter-irritation, M. Bouchardat strongly recommends a plaster which has been much used by M. Chomel at the Hôtel Dieu, and which is thus prepared: Four parts of diachylon-plaster are melted at a very gentle heat, and while it is half liquid one part of croton oil is mixed with it, and the mixture is then spread in a thick layer on calico. Pieces cut from this may be applied to the skin, like ordinary sticking-plaster, and quickly produce an active irritation.—*Ibid., from Bulletin Général de Thérapeutique.* Mars, 1842.

---

*Tannin, as an Antidote for Strychnia.* By Dr. LÜDICKE.—The great care requisite in the employment of strychnia is well known. Of this and of the benefits of tannin when an over dose has been administered the following case is an illustration.

A woman, thirty-seven years of age, had complained for a considerable time of great tenderness and of a wandering pain in the course of the transverse and descending colon, for which opium and various other remedies were unsuccessfully employed. Dr. Lüdicke then ordered one twenty-fourth of a grain of nitrate of strychnia every hour. This dose, however, the patient ventured to increase, and continued to do so till she had taken half a grain of strychnia in the course of six hours. She was now suddenly seized with vertigo, and fell to the ground in a state of insensibility. A quarter of an hour afterwards she complained, though speaking with great difficulty, of having had a sensation as though her spine were being bent backwards, which had then passed off, leaving pain in the back, and tremor of the hands. The respiration was difficult, the pulse feeble and frequent, and the patient had a sensation of nausea with giddiness whenever she attempted to sit upright, and occasional vomiting. Ice was applied to the head, and half a grain of tannic acid was given every hour, at first in an effervescing mixture, on account of the sickness, afterwards in distilled water. After twelve grains had been taken the writer substituted for it a decoction of two ounces of oak bark in six ounces of water, with an ounce of syrup of cinnamon and a scruple of sulphuric æther. The symptoms of poisoning all disappeared, and with them the wandering pains from which the patient had previously suffered.

Mesner, in Dresden, recommends, as an antidote for strychnine, decoction of galls or of oak bark : five ounces of which precipitate two grains of nitrate of strychnine. These decoctions likewise precipitate morphin, codein, brucin, veratrin, and many other vegetable alkaloids.—*Ibid., from Medicinische Zeitung.* Märs 16, 1842.

---

*Ergot of Rye in Uterine Hæmorrhage.* By W. Vellacott, Surgeon R.N.

"Case.—A few weeks ago I was sent for to Mrs. ——, a lady about twenty-six years of age, who had been married a little more than six months, and became pregnant almost immediately after her marriage. I found her labouring under uterine hæmorrhage, which had come on suddenly whilst sitting with some female friends: what quantity of blood was passed before gaining her chamber I do not know ; but *there* I saw about a pint of bright arterial blood, which she said had come away at once, and that it still continued 'to run from her ;' though previously in good health, she appeared to be then, what she always has been in bodily habit, relaxed and delicate ; her pulse was rather accelerated, but small, soft, and feeble ; skin rather warmer than usual ; bowels costive ; in other respects there was nothing deserving of particular notice. On making inquiry I found that about five weeks before she had 'seen a change,' and lost about the same quantity of blood, but never since, until the time I was sent for, although she had continued to walk about as usual. I enjoined perfect rest in the recumbent posture, and gave the following :

℞. Diluted Sulphuric Acid, ʒj.;

Tincture of Opium, m. xl.;

Syrup of Red Poppies, ʒij.,

Water, ℥vss. M.

A fourth part to be taken every four hours.

"I requested her to be kept quiet and cool, and to abstain from animal food, and everything stimulating, and to send for me should the flooding (which became slight before I left her) increase, or any pain come on.

"I saw her the following day, and found her in nearly the same state, having been obliged to have the 'cloth' changed frequently. She now comcomplained of having occasionally 'a slight pain in the bottom of the bowels.'"

As it appeared urgent that labour should come on, Mr. Vellacott attempted to promote labour by pressure on the abdomen. This not succeeding, he commenced the ergot of rye "by administering one drachm of the tincture every fifteen minutes, until pains were produced. The first dose produced a pain, the effect of which was slightly felt at the os uteri, and when four doses had been taken, strong, continued and effectual labour pains came on ; the membranes began to protrude, the mouth of the womb to dilate, and the child's head to descend ; the water soon 'broke,' and the labour was finished in about an hour and a half from my giving the first dose of the ergot tincture, after the taking of which no further hæmorrhage took place, although before that time it had occurred every time she felt 'pain at the bottom of the bowels.' The placenta, which was soon expelled, was found to have at one part a coagulum of dark blood, about three inches long and two wide, evidently the seat of detachment and consequent hæmorrhage; the child was evidently a six months one. It was wrapped in soft warm flannel, being born alive, and continued to live three hours; opened its eyes when a candle was placed before them, and evidently made an effort to swallow when the nurse gave it some sugar and water."—*London Lancet.* June 11th, 1842.

---

*Physiological results of Extirpation of the Salivary Glands.* By Dr. Budge.—Litmus paper introduced into the mouths of a considerable number of rabbits, was changed into a deep blue colour. The same was the result in the case of dogs and cats, without any exception. A cat was deprived of food during two days and a half, and a dog during one day; yet in both cases was the above change in the litmus paper not the less marked. A rabbit and cat had both their nervi vagi cut across: in both animals, till the moment of death, an alkaline reaction of the saliva was manifested. The cat lived till the fourth day. A dog had both its parotid, both its submaxillary, and both its sublingual glands extirpated; yet, to the astonishment of the author, the reaction was still alkaline in as great a degree as before. The glands themselves, after being washed, so as to free them from all traces of blood, were cut into and tested: the reaction was alkaline, but not in so great a degree as when the paper was introduced into the mouth. The animal quite recovered, and during the four weeks which it was permitted to survive, litmus paper introduced into the mouth, was always tinged blue. It was killed, and on examination a small quantity of food was found in its stomach. One bit of litmus paper laid on the stomach remained unaltered in colour; another piece became slightly reddened. The acid reaction seemed less than usual; but the author adds, that in dogs whose glands had not been extirpated he has often noticed very faint traces of acid. The results in a cat, which survived the operation four weeks without the smallest apparent injury, were nearly identical.

From these experiments the author infers that the spleen is not the only gland which can be extirpated without destruction of life. Yet no one can suppose that the salivary glands are useless. We must therefore conjecture that certain glands supplement each other; and that in the case of the removal of the salivary glands the pancreas, perhaps, eliminates the fluid which these glands usually do. It is known for certain that even the urinary secretion cannot remain in the blood when the kidneys are extirpated, and that the stomach endeavours in this case to eliminate, and *does* eliminate, genuine urine, though not, of course, in quantity sufficient for the purposes of life.

Although, according to experiments and observations on the human subject, the saliva, after eating, manifests an alkaline reaction, yet section of the vagus in brutes produces no change. The sympathy between parts supplied by the vagus and those supplied by the trigemini cannot here be taken into consideration. It is possible that this sympathy between the stomach and salivary glands may take place through the spinal branches received by the parotid.—*Brit. and For. Med. Rev.*, *from Medicinische Zeitung*, No. xviii. Mai 4, 1842.

*On External Fistulæ of the Larynx.* By M. Trousseau.—The cases here detailed are the results of inflammation of the submucous tissue of the larynx producing first ossification, and then necrosis of the cartilages. In some cases the matter which is formed makes its way to the interior of the larynx, and being discharged as fast as it is formed the disease goes steadily on till the necrosed portion is separated, and a cicatrix formed over its place. In others, however, matter forms on the outside of the larynx, and opening in the neck a fistula is produced, which either remains permanently open, or closes for a time and then reopens, and so on. Four striking cases unconnected with phthisis are related, and the main practical point deduced from them is, that when there is an external fistula with necrosis no attempts should be made to close it; for the necrosis is never superficial, but extends through the whole thickness of the ossified cartilage, and if the matter be prevented from discharging itself externally it will certainly continue to be formed, and produce much more serious mischief by pressing inwards upon the mucous membrane of the larynx, or by exciting inflammatory swelling about the glottis.—*Ibid.*, *from Journal des Connaissances, Méd. Chir.* Mars, 1842.

---

*Artificial Anus. Closure of the Intestinal Canal by a Tumour in the left Iliac Fossa.* By M. Vidal (de Poitiers.)—This case contains an account of the fifth operation performed by M. Amussat for the formation of a false anus. After frequent postponements of the operation because the intestinal canal did not seem to be completely closed, though the abdomen was tender and excessively distended, it was performed on the 10th day, after nothing but gas had been passed by the anus, and on the 40th from the commencement of the obstruction. The ascending colon was with some difficulty opened, and the patient from that time slowly recovered his health, though the tumour which was supposed to be carcinomatous, remained. Ten weeks after the operation he was in a satisfactory state; he had gained much of his former strength; the artificial anus gave free passage to the fæces, and the discharge of these had become less frequent.—*Ibid.*, *from Gazette des Hôpitaux.* Mars 26, 1842.

---

*On Fractures of the Lower Extremity of the Radius.* By M. Velpeau.—M. Velpeau entirely contradicts Dupuytren's account of the ill consequences of this injury when treated as a sprain or a dislocation, and says, that while he has seen many cases, which thus treated, have recovered with scarcely any deformity and no loss of motion, he has known many more which, though treated carefully with approved apparatus, have presented all the bad results of stiffness of the joint and defective power of the muscles. He believes that all the apparatus hitherto described do more harm than good; and says, the only useful mode of treatment is that with the dextrine bandage. After reducing the fracture he puts a compress, wet with camphorated spirit, round the wrist, and applies a dry bandage very lightly from the roots of the fingers to the middle of the arm. Over this he places graduated compresses reaching to the beginning of the metacarpus, and then an anterior and posterior splint of moistened pasteboard, which are moulded exactly on the parts they have to cover, and descend to the roots of the fingers. A bandage wet with starch is then rolled in a double layer, from the fingers to a short distance above the elbow; and, till it has dried, all the parts are kept in their places by two long wooden splints. These last, however, are removed after six and eight hours, and the part left in its immoveable bandage supported in a sling, for from twenty to thirty days, by which time the union of the fracture is generally perfected. There are but few cases, M. Velpeau adds, in which this method of treatment is not sufficient.—*Ibid.*, *from Ibid.* Janvier 13, 1842.

---

*Statistical Researches into the Etiology of Pulmonary Phthisis.* By Dr. Briquet, of the Hôpital Cochin.—This paper is founded on an investigation into various particulars connected with the history of one hundred and nine phthisical patients in whom the disease was far advanced, and likewise on data furnished by all the deaths from phthisis in the hospital between January 1st, 1838, and January 1st, 1841, being one hundred and eighty-two in number.

The conclusions (for which only we have space,) at which M. Briquet arrives are:

1. That during the past three years one-tenth more of men than of women have been received into the Hôpital Cochin affected with phthisis: a result directly contrary to those obtained by MM. Lombard and Louis.

2. In at least a third of the patients phthisis was distinctly hereditary, and predisposition to the disease seemed more frequently to come from the father than the mother.

3. No immunity from the disease is afforded by the circumstance of being born of parents who are natives of the country, or by being brought up in the country.

4. Tall stature, a slender frame, an ill formed chest, and convexity from the root to the point of the nails are the only external characteristics of phthisical diathesis.

5. It occurred very seldom that the circumference of the upper part of the chest was less than that of the lower part; a fact directly contrary to the assertion of M. Hertz.

6. Those callings in the pursuit of which there is discomfort, want of exercise and of pure air present a greater number of phthisical persons than is to be found among those who pursue different occupations.

7. A third of these patients were more subject to catarrh than other persons, and were more sensible of cold.

8. In three-fifths of the patients phthisis developed itself between twenty and thirty years of age, but more than two-thirds of those whose parents had suffered from consumption became phthisical before their thirtieth year; while, of those whose parents had not been healthy, half did not show symptoms of phthisis till after thirty.

9. In four-fifths of the patients there existed predisposition to phthisis, and in three-fifths this predisposition was acquired.

10. Cold is the most powerful cause of the acquired predisposition; next to which are misery, privation, and distress of mind.

11. Phthisis is most frequent in cold seasons, and when there are many variations in the atmosphere.

12. Four-tenths of the patients had not been exposed to the influence of any occasional cause of phthisis, but in most there existed a strong predisposition to the disease.

Five-tenths had been exposed to and suffered greatly from some exciting cause, and this cause was in almost every instance cold and damp.—*Ibid.*, *from Revue Médicale.* Feb. 1842.

---

*Injections of the Nitrate of Silver.*—Dr. Daniel, of Cetto, describes the case of a Venetian, thirty-two years old, labouring under catarrh of the bladder, complicated with syphilis, impotence, and more or less involuntary discharge of semen every morning. The syphilitic complaint was treated with baths containing the deuto-chloride of mercury, and for the affection of the bladder and caput galinaginis he was ordered an injection of 125 scruples of distilled water, in which had been dissolved 32 grains of nitrate of silver. This was injected into the bladder with the best effects. The strength of the solution was gradually increased, and the patient was cured in less than three months of his syphilis, vesical catarrh, and impotence. Dr. Daniel heard afterwards that he had recovered, and that his wife was pregnant.—*Provincial Med. Journal.* June 11, 1842. *From Journ. de Med. Pratique de Montpellier.*

---

*Introduction of Air into Veins.*—Dr. G. Cappelletti, of Trieste, has published two cases of this accident, which is in general so fatal, but which was, in these instances, followed by recovery. The first case was that of a widow lady affected with ulcerated cancer of the breast, a relapse after two previous operations, and which Dr. Cappelletti removed, at the earnest request of the patient. The cancer extended to the armpit, but the glands in the axilla were not diseased. While the adhesions near the hollow of the axilla were being divided, and a portion of the pectoralis major, which was diseased, was being removed, a peculiar sound was heard, at first supposed to be caused by the hardness and resistance of the muscular fibre, but which was soon recognised as the peculiar hissing noise described by Amussat. Scarcely was it heard when the patient sighed deeply, exclaimed that she was dying, and fell almost lifeless into the arms of a surgeon who was present. A sponge was immediately applied to the wound, in order to prevent, by moderate pressure, the admission of any more air. The pulse was frequent, irregular, and intermittent; respiration difficult; the eyes half closed; and the patient from time to time applied her hand to the region of the heart, taking frequent and painful inspirations. The remedies usually employed in cases of syncope were had recourse to, and, contrary to expectation, the pulse became gradually more regular, respiration less laborious, and in less than ten minutes all the alarming symptoms had disappeared. The patient got well, and had not had a relapse of the cancer at the date of the report, more than a year after the operation. The patient described her sensations during the alarming condition to which she was reduced as follows:—She was not at any time entirely deprived of the use of her senses, but was utterly incapable of speaking a word; she felt an acute pain at the heart, as if it were about to cease to beat, and as if she must die.

In the second case, Professor Koepl, senior surgeon of the hospital at Trieste, was removing a large medullary fungus from the cheek of a man, fifty years old, which extended down to the neck, and had nearly completed the operation, when the peculiar pathognomonic hissing sound was heard, and the patient, uttering a sharp cry, dropped, as if struck by lightning. The cause was evident, the entry of air by the external jugular vein. Compression was immediately applied over the wounded vessel, and a ligature thrown around the tumour. The patient recovered in ten minutes from this dreadful state, contrary to the anticipation of the operator, who fully expected his death.—*Ibid., from Annali Universali di Medicina.*

---

*Tincture of Sesquichloride of Iron in Gleet.* By S. E. R. Jones, Surgeon.—In former years I have had considerable difficulty in the thorough cure of "*gleet*," being able to put an end to the discharge for a time, as is generally the case, to return.

I will now relate "two cases" out of many where I have succeeded in permanently eradicating this troublesome complaint by the tincture of sesquichloride of iron.

John Thomson, aged 24, had gonorrhœa eighteen months ago, and gleet continued since; has been under treatment of a surgeon, who ordered cubebs, copaiva, &c., but as he was careless of consequences did not apply regularly: he had a discharge of thin, white pus when he applied to me. Ordered nitrate of silver and sulphate of zinc injections, alternately to be used, but without effect. I then made use of tincture of iron, gts. xxv., three times a-day, and gradually increased to gts. xl.; in a fortnight he was perfectly well; has had no return since.

Wm. Simpson, aged 21, had gleet twelve months; ordered the tincture of iron, dose, gts. xx., two or three times a-day; to be increased to dose gts. xxxv. After seven or eight days the discharge had nearly ceased; in fifteen days he was cured.—*London Lancet.* July 2, 1842.

---

*Musk.*—Dr. Hanle, of Lahr, prepared a mixture containing six grains of musk, three ounces of berry laurel-water, and six drachms of syrup of almonds, and sent it to the patient for whom it was prescribed. To his great surprise it was returned to him, because it did not possess the odour of musk, and either contained very little, or else an inferior article. Dr. Hanle, well aware that he had used the best musk, ascertained by experiment that the odor was changed by the addition of the syrup of almonds. Messrs. Soubeiran and Boudet followed in his steps, confirmed his statement, and discovered further that the same effect is produced on assafœtida. The odour of six grains of musk is so much diminished by forty scruples of the syrup of almonds, that some persons, not aware of the experiment, could not detect it, while others discovered it, but very feebly.—*Provincial Medical Journal, from Bulletin de Thérap.*

---

*Inflammation of the Cœcum—Accumulation of Fæces.*—Dr. Gerbaud has published several cases of inflammation of the cœcum, caused by the accumulation and induration of fæcal matter in the intestines. In one of these cases the diagnosis was verified by the autopsy; the cœcum was found to be the only part inflamed, and its mucous membrane thickened, softened and ulcerated. Inflammation of the cœcum comes on suddenly, and the seat and intensity of the pain aid the diagnosis materially. The symptoms resemble those of hernia, local peritonitis, and acute cystitis, but its situation and progress will distinguish it from those affections. A long circumscribed tumor in the situation of the cœcum is found in those cases where the inflammation is caused by the fæcal accumulation, and is not unfrequently mistaken for other diseases. Lisfranc was sent for, fifteen years ago, to a patient who was said to have a fibrous tumor in the rectum. She was suffering from diarrhœa, and no one suspected the accumulation of fæces in the intestine. She was paraplegic, and the orifice of the rectum being very dilatable, a tumor, covered with a reddish pellicle, could be easily distinguished. On examination, and scratching this with the nail, Lisfranc discovered, to his surprise, that it consisted of a fæcal mass enveloped in false membranes. It was extracted, but not without difficulty.

Retention of stercoral matter in the lower portion of the intestinal tube, as high up as the sigmoid flexure, is of more frequent occurrence than is supposed. Authors advise the extraction of these matters with a curette, when they are within reach, but Lisfranc considers that dangerous consequences might arise from such a proceeding. He recommends, in preference, breaking them up with the index finger, and washing them out with injections; this, however, is not always an easy operation, on account of their being situated in a distant part of the intestine.—*Prov. Journ. June* 25, *from Lond. and Ed. Monthly Med. Journ.*

[*Remarks.*—Great difficulty is often experienced, in cases of impacted fæces in the rectum, from the reagglutination of the mass, after being partially broken up by the finger or spoon; nor is it possible safely to introduce the former in all cases, owing to the extreme dryness of the fæces. We have experienced the happiest effects from the introduction of a small, damp suppository of some mild soap in the first instance, followed, after twenty or thirty minutes, by an injection of one or two ounces of warm water, to facilitate the introduction of the finger, and to prevent—which it will most effectually do—the coalescence of portions detached by its means, or by the spoon. After the removal of the portion of the mass nearest the anus, which is then very easily effected, nothing more powerfully promotes the softening and partial solution of the remains, than injections of warm, weak castile soap-suds.

The greatest obstacle to the natural evacuation, in such cases, is the extreme dryness, not only of the fæces, but of the irritated mucous surface, in contact with them. Even when temporarily paralysed by distention, we have generally seen the rectum speedily recover its contractile power under this bland application, rendering further mechanical relief unnecessary.]

R. C.

---

The following case is given, by its author, in the Provincial Journal for June 18th, 1842, as an additional proof that complete luxations of the knee joint, do not on all occasions involve the necessity of amputation. The paper which contains it, was elicited by the history of a recent case by Mr. Coote, to which we have referred at page 533.

*Case of Complete Dislocation of the Tibia forwards.* By Jonathan Toogood, Esq., Senior Surgeon to the Bridgwater Infirmary.—Complete dislocation of the knee joint is so very rare an accident, that I believe few surgeons have ever seen it. I furnished Sir Astley Cooper with the following case, which he published in his work on Dislocations. He had never met with a similar one, nor has any surgeon of my acquaintance, connected with the London or provincial hospitals, although I have related it to a considerable number; neither do I remember to have read of such a case. I learnt also, in a conversation with Dupuytren and Roux, that it had not occurred in their practice.

Dec. 5, 1806. Francis Newton, a strong, athletic man, thirty years old, fell from the fore part of a waggon heavily laden with coals, and, entangling his foot in the frame work of the shaft, was dragged a very great distance before he was released. I saw him two hours after the accident; the left knee was very much swollen; the tibia, fibula, and patella were driven up in front of the thigh, and the os femoris occupied the upper part of the calf of the leg, the internal condyle being nearly through the skin. It was a complete dislocation, and the appearance of the limb was so dreadful that I despaired of being able to reduce it; but to my surprise it was more readily effected than I imagined. By placing two men at the thigh whilst I extended the leg, the reduction was immediately effected. The whole limb was placed in splints, and the strictest antiphlogistic treatment observed, with the most perfect quiet. The symptoms were very mild, and, by carefully watching him, he suffered very little inflammation or pain. At the expiration of a month I allowed him to get up, and on the 29th of January he came into this town, a distance of four miles, in a cart, and walked from an inn to my house, with his leg but little swollen, and having some motion of the joint. He eventually recovered very good use of his limb, and walks with so little inconvenience, that he has followed his business as a waggoner ever since, and this day (November 30, 1822) I have seen him walking by the side of his team with very little lameness.—*Prov. Med. Journ.*, June 18, 1942.

---

*Case of Partial Dislocation of the Tibia, outwards.* By J. Ballard Pitt, Surgeon. Mattishall, Norfolk, June 7, 1842. (Condensed.)—A young lady, on the 2nd of February, produced partial lateral dislocation of the right knee-joint, by falling down stairs with great violence; she was immediately carried to her bed-room, and laid upon a mattrass. On examination I found the internal condyle of the femur projecting considerably inwards, and the external tuberosity of the tibia outwards; she shrieked violently, and complained of severe pain, similar to cramp, down the outer side of the leg. Having placed the patient on her back, I made gradual extension and counter-extension, which, together, soon brought the joint into its former position; lateral splints were then put on, and bandaged in such a manner, as to allow leeches and lotions to be applied. The following draught was taken at bed-time.—Tincture of hyosciamus, half a drachm; water, ten drachms; Syrup of roses, one drachm.

Feb. 3. Passed a restless night; considerable swelling of the joint, with much pain and tenderness, particularly along the course of the internal lateral ligament. Eighteen leeches to be applied, and evaporating lotions, and to take the following draught:—Sulphate of magnesia, six drachms; Carbonate of magnesia, half a drachm; Infusion of senna, one ounce and a half; Tincture of senna, one drachm.

4. Bowels relieved; did not sleep much; swelling increased. Twenty leeches to be applied, and lotion continued.

5. Complains of pain, extending up the thigh; bandages loosened; great heat of the joint; swelling and tenderness the same; and a sensation of effusion upon pressing down the patella. Twelve leeches applied, and to take the following pill and draught:—Opium, one grain; Calomel, three grains. An aperient draught in the morning.

From the 6th to the 9th, the leeches and fomentations were continued, but the details are unimportant.

From the 9th till the 15th nothing particular occurred; swelling and pain decreased; and on the 16th I removed the splints, fomented the joint, and made a little passive motion, which caused severe pain on its inner side, and a sensation of stiffness over the centre.

17. Passive motion repeated.

19. Placed the leg over a double inclined plane for a short time. From this date perfect rest was allowed till the 25th, when a little flexion was again tried, and borne with much less suffering. After this period, a little linim. saponis co. was rubbed in daily, and she was put to sit upon the edge of a table, and made to bend and extend the limb several times, which gradually gained strength and freedom of motion. She now protects the joint with an elastic knee piece, and is able to walk comfortably.—*Prov. Med. Journ.*, June 18, 1842.

---

*On a peculiar inflammatory affection of the Cornea in Nurses.* By Professor Nasse.—A malignant form of keratitis or inflammation of the cornea occasionally accompanies puerperal attacks, and in general terminates fatally. This affection, however, is not of a malignant nature, and appears at any time during the whole period of nursing frem a month after delivery to a year and a half, if the child be suckled so long. The eye is felt irritable and the conjunctiva is seen injected with blood. Occasionally the catarrhal symptoms attend the complaint, at other times little vesicles appear over the surface of the conjunctiva. Sometimes rheumatic symptoms are present, at other times it comes on with a vesicular cutaneous eruption over the face. The conjunctival inflammation rapidly passes to the cornea and is accompanied by the usual darting pains in the eye and margin of the orbit. From the third to the eighth day an abscess forms within the layers of the cornea, when the inflammatory symptoms diminish, and, if nothing be done to put an end to the complaint, it bursts into the anterior chamber and occasions hypopion.

The disease is not peculiar to any age, constitution, or season; but is in every case preceded by great lassitude, debility, and leanness, brought on by excessive lactation, in fact, seems to be a disease of debility. Blood-letting is consequently never indicated, but blisters behind the ears, diaphoretics combined with bitter infusions, quinine and sulphuric acid, a tonic diet, and above all, the giving up suckling the child, generally effect a cure in about three weeks. It is mentioned that the separation of the child is the most important part of the treatment; and cases are related where the child being allowed to suckle before the cure was completed, brought it back with increased severity, and could not be stopped till the child was again removed, when the disease rapidly gave way.—*Ed. Med. and Surg. Journ., from Ammon's Monatschrift far Medicin.*

